# To Keep Ice

**Published:** 1875-04

**Authors:** 


To KEEP ICE.—Make a double pocket of strong woolen cloth, no matter how coarse and faded it is. Have a space about two inches or so between the inner and outer pockets, and pack this space as full as possible with feathers. You have no need to use geese feathers; hen feathers are just as good. With a pocket thus constructed and kept closely tied at the mouth, a few pounds of ice may be kept a week.

